# Molecular and pharmacodynamic insights into β-lactam therapy for high-inoculum *Enterobacter cloacae* complex infections

**DOI:** 10.1128/aac.01170-25

**Published:** 2025-09-22

**Authors:** Ashlan J. Kunz Coyne, Rachel Gray, Elizabeth May, Hunter Curry, Nicole Slain, Chris Delcher, Eugene Shin

**Affiliations:** 1 University of Kentucky College of Pharmacy 15511, Lexington, Kentucky, USA; 2 University of Kentucky HealthCare 177468, Lexington, Kentucky, USA; 3 Institute for Pharmaceutical Outcomes and Policy, University of Kentucky College of Pharmacy 15511, Lexington, Kentucky, USA; Johns Hopkins University School of Medicine, Baltimore, Maryland, USA

**Keywords:** *Enterobacter cloacae *complex, AmpC-producing *Enterobacterales*, β-lactamase expression, β-lactam/β-lactamase inhibitors, inoculum effect, simulated endocardial vegetation model

## Abstract

High-inoculum *Enterobacter cloacae* complex (ECC) infections challenge β-lactam therapy due to β-lactamase-mediated resistance and the inoculum effect, where high bacterial densities amplify resistance and worsen outcomes. We evaluated the pharmacodynamic efficacy of cefepime, meropenem, ertapenem, meropenem–vaborbactam, and ceftazidime–avibactam against three high-inoculum (10⁹ colony-forming unit [CFU]/g) ECC isolates (0008, 0032, and 0060) harboring diverse β-lactamases using a 96 h simulated endocardial vegetation (SEV) model with humanized exposures. Bactericidal activity, resistance emergence, and β-lactamase expression were assessed. Cefepime failed in all isolates, with SEV growth from +0.4 to +1.6 log_10_ CFU/g and minimum inhibitory concentrations (MICs) increasing from 2–8 µg/mL to >64  µg/mL in 0008, 0032, and 0060, coinciding with AmpC β-lactamase (ACT) induction up to 16.1-fold (0032). In 0060, cefepime failed, whereas meropenem displayed infusion-dependent efficacy: the 3 h infusion achieved bactericidal killing (−4.6 log_10_ CFU/g), while the 30 min infusion produced partial killing (–2.9 log_10_ CFU/g). Meropenem failed in 0032 (+1.1 log_10_ CFU/g), with ACT-16 induction reaching 14.6-fold. Ertapenem produced initial bactericidal activity in 0060 (–3.2 log_10_ CFU/g) but regrowth to –1.9 log_10_ CFU/g by 96 h, accompanied by ACT-89 expression rising 26-fold. Meropenem–vaborbactam was bactericidal in 0008 (–4.6 log_10_ CFU/g) and 0032 (–3.6 log_10_ CFU/g) with stable MICs and minimal β-lactamase induction. Ceftazidime–avibactam achieved bactericidal activity in 0008 (–4.9 log_10_ CFU/g) but only bacteriostatic activity in 0032 (–1.2 log_10_ CFU/g), with modest KPC-3 and ACT-16 upregulation (~1.7- to 2.3-fold). β-Lactamase diversity and inoculum effects limit β-lactam efficacy in high-inoculum ECC infections. Meropenem–vaborbactam showed consistent activity, and prolonged meropenem infusion benefited select isolates. Variable ceftazidime–avibactam efficacy reinforces aligning β-lactam selection with underlying β-lactamase mechanisms.

## INTRODUCTION

The *Enterobacter cloacae* complex (ECC), a key AmpC-producing *Enterobacterales* (AmpC-E) alongside *Klebsiella aerogenes* and *Citrobacter freundii*, is a significant opportunistic pathogen in nosocomial infections (1, 2). Although ECC endocarditis is uncommon, its occurrence is associated with high mortality, particularly due to the high bacterial burden typical of deep-seated infections, where treatment outcomes are poor (3–5). In such infections, the inoculum effect, a phenomenon where high bacterial density enhances resistance mechanisms, can exacerbate β-lactam failure through increased β-lactamase expression, porin downregulation, efflux pump activity, and biofilm formation, even when standard inoculum minimum inhibitory concentrations (MICs) suggest susceptibility (6–9).

Standard susceptibility testing (10⁵–10⁶ colony-forming units [CFU]/mL) often fails to predict outcomes in infections with high bacterial burdens. Consequently, ex vivo pharmacokinetic (PK)/pharmacodynamic (PD) models have become essential for evaluating antibiotic efficacy under clinically relevant conditions (7, 10, 11). β-Lactam antibiotics, including cephalosporins (e.g., cefepime), carbapenems (e.g., meropenem and ertapenem), and β-lactam/β-lactamase inhibitor (BL/BLI) combinations (e.g., meropenem–vaborbactam and ceftazidime–avibactam), remain vital in treating ECC infections (12), yet their reliability is increasingly challenged by inoculum-dependent resistance mechanisms (13).

Cefepime activity is particularly compromised under high-inoculum conditions, with MIC_90_ values rising from 8 to 256 µg/mL and resistance rates increasing from 6.5% to over 50.0% (14). This effect is largely driven by elevated AmpC β-lactamase (ACT) expression, independent of sequence type or AmpC variant, and amplified during therapy (15, 16). Although cefepime is considered a carbapenem-sparing option and has support from retrospective ECC bacteremia studies, its reliability in high-inoculum infections, especially for susceptible dose-dependent (SDD) isolates with MICs of 4–8 µg/mL, remains uncertain due to its susceptibility to AmpC-mediated hydrolysis (2, 15, 17–20). Notably, isolates with baseline cefepime MICs of ≥1 µg/mL are more likely to develop resistance under high bacterial burden due to increased β-lactamase activity, even in the absence of porin mutations or other structural resistance mechanisms (14).

Acquired β-lactamases, including *Klebsiella pneumoniae* carbapenemase-3 (KPC-3) and the class A enzyme TEM-1, markedly restrict therapeutic options by compromising carbapenem activity (21–23). The interplay between β-lactamase induction, porin loss, and efflux pump activity contributes to treatment failures despite susceptible MICs, highlighting the disconnect between *in vitro* susceptibility and *in vivo* efficacy in high-inoculum settings (24, 25). Newer BL/BLI combinations, such as meropenem–vaborbactam and ceftazidime–avibactam, target KPC and some AmpC enzymes, but their performance in high-inoculum infections remains inadequately studied (26–29). High bacterial loads reduce antibiotic penetration into biofilm-like vegetations, amplify resistance mechanisms, and promote adaptive resistance during prolonged β-lactam exposure (8, 30–32). While extended infusion regimens may optimize time-dependent killing, their effectiveness in deep-seated ECC infections is uncertain (33). Current Clinical and Laboratory Standards Institute (CLSI) testing methods, designed around standard inoculum models, fail to detect dynamic resistance phenomena such as inoculum effects, underscoring the importance of advanced ex vivo models to simulate clinically relevant conditions (6, 24, 25, 34).

Given these challenges, this study evaluates the PD efficacy of β-lactam regimens against high-inoculum ECC isolates harboring ACT variants, KPC-3, and TEM-1 while examining dynamic changes in β-lactamase expression. We employed a 96 h simulated endocardial vegetation (SEV) model with humanized antibiotic exposures to evaluate cefepime, meropenem, and ertapenem, which are current standards of care for AmpC-E, as well as the BL/BLI combinations meropenem–vaborbactam and ceftazidime–avibactam, both designed to overcome KPC-mediated resistance. Cefepime was included to evaluate its carbapenem-sparing potential in high-inoculum settings despite its vulnerability to AmpC-mediated hydrolysis, whereas meropenem and ertapenem were selected to compare carbapenem efficacy across infusion durations and β-lactamase profiles. Meropenem–vaborbactam and ceftazidime–avibactam were assessed for their ability to overcome KPC- and AmpC-driven resistance, as their performance in high-inoculum ECC infections remains poorly characterized despite their clinical activity against resistant *Enterobacterales*. This model provides a platform to compare both traditional and novel therapies under conditions that closely simulate deep-seated infections such as endocarditis (35, 36).

## MATERIALS AND METHODS

### Bacterial isolate selection

Three well-characterized ECC isolates were evaluated using 96 h ex vivo PK/PD models with a targeted starting inoculum of 10⁹ CFU/g. The isolates (0008, 0032, and 0060) were obtained from the Centers for Disease Control and Prevention and Food and Drug Administration Antimicrobial Resistance Isolate Bank. Phenotypic and genotypic characteristics for each isolate are listed in Table 1. These isolates represent a clinically relevant spectrum of resistance mechanisms, including ceftriaxone resistance, cefepime SDD and susceptible phenotypes, and both carbapenem-susceptible and carbapenem-resistant profiles. Additionally, they harbor diverse AmpC β-lactamases, including ACT variants, among the most commonly identified in ECC isolates in the United States, along with other clinically significant β-lactamases, such as KPC-3 and TEM-1 (37).

**TABLE 1:** *Enterobacter cloacae* phenotypic and genotypic characteristics^*b*^

AR CDC number	MIC (μg/mL)^*a*^					β-Lactamase genes
	Cefepime	Meropenem	Ertapenem	Meropenem–vaborbactam	Ceftazidime–avibactam	
0008						ACT-15
5 × 10^5^	8.0	2.0	>8.0	≤0.5	1.0	
5 × 10^7^	≥1,024	NA	NA	≥1,024	≥1,024	
0032						ACT-16, KPC-3, and TEM-1
5 × 10^5^	8.0	0.25	1.0	≤0.5	≤0.5	
5 × 10^7^	≥1,024	≥256	NA	≥512	≥128	
0060						ACT-89
5 × 10^5^	2.0	≤0.12	0.25	NA	NA	
5 × 10^7^	≥256	≥128	≥256	NA	NA	

^*a*^ MICs were tested at standard (5 × 10^5^ CFU/mL) and high (5 × 10^7^ CFU/mL) inocula at baseline to assess the inoculum effect. Antibiotics not tested against specific isolates in the ex vivo models are indicated as NA in the table.

^*b*^ ACT, AmpC-type βlactamse; AR, antibiotic resistance; CDC, Centers for Disease Control and Prevention; KPC, *Klebsiella pneumoniae* carbapenemase; MIC, minimum inhibitory concentration .

The ECC isolates were cultured on tryptic soy agar (TSA) plates and incubated at 37°C for 24 h prior to evaluations. Mueller–Hinton broth (Millipore Sigma, Darmstadt, Germany) supplemented with calcium and magnesium at concentrations of 25.0 and 12.5 µg/mL (CAMHB), respectively, was used for 96 h models. Colony counts were performed on TSA plates (Hardy Diagnostics, Santa Maria, CA, USA).

Minimum inhibitory concentrations of study antimicrobials were determined in triplicate by manual broth microdilution (BMD) using a 96-well plate format at standard inoculum (10⁶ CFU/mL) per CLSI protocol (6) and at higher inoculum (5 × 10⁷ CFU/mL) to evaluate the inoculum effect at baseline. Quality control (QC) strains *Escherichia coli* American Type Culture Collection (ATCC) 25922 and *Klebsiella pneumoniae* ATCC 700603 were included in each run, as appropriate, and all MIC values were verified to be within CLSI-defined QC ranges. The higher inoculum was not tested for SEV model samples. For BMD MIC assays, the modal MIC (most frequent value across replicates) was reported. Only β-lactams demonstrating susceptibility at baseline at standard inoculum were advanced for evaluation in the SEV models.

Antimicrobials tested include cefepime (CHEM-IMPEX, lot 002179-300803), meropenem (Matric Scientific, lot 075811), ertapenem (Research Products International, lot 200214-200251), meropenem–vaborbactam (Melinta Therapeutics, lot 007E2), and ceftazidime–avibactam (AbbVie lot 24K03221).

### Ex vivo PK/PD model

Ex vivo SEV PK/PD models were conducted in duplicate, with SEV clots prepared by mixing ECC isolates with human cryoprecipitate and platelets (Kentucky Blood Center), combined with fibrinogen and activated with thrombin to mimic endocardial vegetations, as previously described (38–41). Models were incubated at 37 °C for 96 h, with CAMHB circulated using a peristaltic pump (Masterflex; Cole-Palmer Instrument Company, Chicago, IL, USA) to simulate antibiotic half-lives. Antimicrobial regimens (Table 2) were humanized to reproduce steady-state exposures observed in adults with normal renal function. In the ertapenem model, the central compartment was prefilled with growth medium supplemented with 3.5 g/dL human albumin to account for its high protein binding (42, 43).

**TABLE 2:** Pharmacokinetic target parameters in 96 h ex vivo models^*c*^

β-Lactam regimen	Target^*a*^		
	fC_max_^*b*^ (µg/mL)	*t*1/2 (h)	fT > MIC (%)
Cefepime 2 g q8h (30 min infusion)	134.4	2	≥60
Cefepime 2 g q8h (3 h infusion)	61.5	2	
Meropenem 2 g q8h (30 min infusion)	96.0	1	≥40
Meropenem 2 g q8h (3 h infusion)	68.5	1	
Ertapenem 1 g q24h (30 min infusion)	15.5	4	
Meropenem–vaborbactam 4 g q8h (3 h infusion)	Meropenem: 43.1 Vaborbactam: 37.5	Meropenem: 1.22 Vaborbactam: 1.68	Meropenem: ≥40 Vaborbactam: ≥30 (fT > *C*_*T*_ 0.5)
Ceftazidime–avibactam 2.5 g q8h (2 h infusion)	Ceftazidime: 82.8 Avibactam: 13.8	Ceftazidime: 2.76 Avibactam: 2.71	Ceftazidime: ≥60 Avibactam: ≥50 (fT > *C*_*T*_ 1.0)

^*a*^ Target pharmacokinetic parameters (fC_max_, *t*1/2, fT > MIC, and fT > *C*_*T*_) were obtained from steady-state drug data reported in the literature and respective package inserts.

^*b*^ Protein binding (%): cefepime 20, meropenem 2, ertapenem 92, vaborbactam 33, ceftazidime 8, and avibactam 8.

^*c*^ fC_max_, maximum observed concentration; fT > CT, percentage of the dosing interval that free drug concentrations exceeded the β-lactamase inhibitor threshold concentration; fT > MIC, percentage of the dosing interval that free drug concentrations exceeded the MIC; *t*1/2, half-life.

Each simulated endocardial vegetation model was run in duplicate, and duplicate samples were collected from each model at 0, 4, 8, 24, 32, 48, 56, 72, 80, and 96 h, yielding a total of four replicates per sampling time point. Each SEV clot was homogenized and centrifuged twice, with supernatant replaced by normal saline after each cycle to remove residual drug. Additionally, 1 mL of broth from the central compartment was collected at each time point to assess bacterial suppression outside the clot. Bactericidal activity was defined as a ≥3-log_10_ reduction in bacterial burden from baseline within the 96 h model. Bacterial counts (CFU/g and CFU/mL) were reported as mean ± standard deviation from duplicate samples. Regrowth was defined as any increase in bacterial burden following initial bactericidal activity. Comparisons of log_10_ CFU/g reductions across regimens were analyzed by one-way analysis of variance with post hoc Tukey’s test, with significance defined as *P* < 0.05.

### Pharmacokinetic analysis

Pharmacokinetic samples were collected in duplicate from the SEV model central compartment at 0, 0.5, 2.0, 3.0, 4.0, 8.0, 24.0, 32.0, 48.0, 56.0, 72.0, 80.0, and 96.0 h, stored at −80°C, and quantified via high-performance liquid chromatography–tandem mass spectrometry (44–48). Steady-state PK parameters (*t*1/2, fC_max_, and % fT > MIC or % fT > CT) were calculated using non-compartmental analysis in Phoenix WinNonlin (version 8.5; Certara, Princeton, NJ, USA). fC_max_ was determined as the maximum observed free concentration; *t*1/2 was calculated from the terminal log-linear phase; and % fT > MIC (for β-lactams) or % fT > CT (for β-lactamase inhibitors) was computed by determining the percentage of the dosing interval that free concentrations exceeded the MIC or threshold concentration, adjusted for protein binding (Table 2) (49–55). Model performance was verified by ensuring mean *t*1/2 and fC_max_ from duplicate samples were within ±10% of steady-state human literature values (Tables 2 and 3).

**TABLE 3:** Achieved pharmacokinetic parameters in 96 h ex vivo models^*c*^

Regimen	Achieved^*a*^		
	fC_max_ (µg/mL)	*t*1/2 (h)	% fT > MIC^*b*^
Cefepime 2 g q8h (30 min infusion)	135.4	2.2	≥99.9
Cefepime 2 g q8h (3 h infusion)	60.8	2.1	≥99.7
Meropenem 2 g q8h (30 min infusion)	103.9	1.1	100
Meropenem 2 g q8h (3 h infusion)	66.8	1.02	100
Ertapenem 1 g q24h (30 min infusion)	16.3	4.06	99.9
Meropenem–vaborbactam 4 g q8h (3 h infusion)	Meropenem: 47.1 Vaborbactam: 41.1	Meropenem: 1.33 Vaborbactam: 1.69	Meropenem: 99.9 Vaborbactam: 100 (*f*T > *C*_*T*_ 0.5)
Ceftazidime–avibactam 2.5 g q8h (2 h infusion)	Ceftazidime: 81.2 Avibactam: 14.3	Ceftazidime: 2.77 Avibactam: 2.52	Ceftazidime: 100 Avibactam: ≥99.9 (fT > *C*_*T*_ 1.0)

^*a*^ All pharmacokinetic/pharmacodynamic indices were calculated using free (unbound) drug concentrations. Protein binding (%): cefepime 20, meropenem 2, ertapenem 92, vaborbactam 33, ceftazidime 8, and avibactam 8.

^*b*^ Percent fT > MIC (or fT > CT) values were calculated using the highest MIC (or CT) observed for the tested isolate–antibiotic combination.

^*c*^ fC_max_, maximum observed concentration; fT > CT, percentage of the dosing interval that free drug concentrations exceeded the β-lactamase inhibitor threshold concentration; fT > MIC, percentage of the dosing interval that free drug concentrations exceeded the MIC; *t*1/2, half-life.

### Resistance development

The development of antibiotic resistance compared to baseline was evaluated in each clot sample using antibiotic-embedded agar and standard inoculum BMD. For antibiotic-embedded agar, we plated 100 µL of the 96 h SEV samples onto individual TSA plates containing threefold the baseline MIC of the tested drug in the model (34, 56). Plates were examined for growth after 24 and 48 h of incubation at 37°C. For BMD, we evaluated 96 h SEV sample antibiotic MICs according to the CLSI protocol (34). For either assay, if samples demonstrated MIC changes of ≥2 doubling dilutions from baseline (elevation or reduction in MIC), then they were passed for three consecutive days of MIC testing. For samples maintaining the ≥2 doubling dilution change in MIC from baseline following the 3-day pass, additional SEV samples were assessed for resistance in a backward stepwise manner from 80 h to earlier time points until a ≤1 dilution change in MIC was identified for the sample.

### β-Lactamase expression

To evaluate β-lactamase-mediated resistance, gene expression was quantified over time by quantitative PCR (qPCR). Total RNA was extracted from 1 mL SEV specimens collected at T0, T24, T48, T72, and T96. Immediately after collection, the specimens were mixed with RNAprotect Bacteria Reagent (Qiagen, cat. no. 76506) to stabilize RNA. Samples were pelleted by centrifugation (5,000 × *g* for 5 min at 4°C) and resuspended in TE buffer. RNA was extracted using the RNeasy Mini Kit (Qiagen, cat. no. 74106) following the manufacturer protocol, with on-column DNase I treatment (RNase-Free DNase Set, cat. no. 79254; Qiagen) to remove genomic DNA. Bacterial cells were lysed with buffer RLT containing β-mercaptoethanol, mixed with 70% ethanol, transferred to RNeasy spin columns, washed with buffer RW1 and buffer RPE, and eluted in RNase-free water. RNA purity and concentration were confirmed using a NanoDrop spectrophotometer (*A*260/*A*280 ~2.0, *A*260/*A*230 >1.8). Purified RNA was stored at −80°C until complementary DNA (cDNA) synthesis.

Complementary DNA was synthesized using the High-Capacity cDNA Reverse Transcription Kit (Applied Biosystems, cat. no. 4374966). Reactions (20  µL) contained 2 µL 10× reverse transcription (RT) buffer, 0.8 µL 25× deoxynucleotide triphosphate mix, 2 µL 10× RT random primers, 1 µL reverse transcriptase, 200 ng RNA, and nuclease-free water. Reverse transcription was performed at 37°C for 120 min, followed by enzyme inactivation at 85°C for 5 min. No-reverse-transcriptase and no-template controls were included to verify the absence of genomic DNA contamination and rule out non-specific amplification. Synthesized cDNA was stored at −20°C and used within 1 week for qPCR analysis.

qPCR was performed on the QuantStudio system using TaqMan Fast Advanced Master Mix (Applied Biosystems, cat. no. 4444963) and custom oligonucleotide primers and probes designed for each β-lactamase gene (Integrated DNA Technologies, Coralville, IA, USA) (Table S1), incorporating gBlock gene fragments for enhanced specificity. Primer and probe sequences were designed using Primer3, validated for specificity via BLAST, and confirmed to have amplification efficiencies of 90%–110% (*R*²  >0.98) using standard curves with 10-fold cDNA dilutions. Reactions (10 µL) contained 5 µL TaqMan Fast Advanced Master Mix, 0.4 µL each primer (10 µM), 0.2 µL probe (10 µM), 1 µL cDNA, and nuclease-free water. Cycling conditions were 95°C for 2 min, followed by 40 cycles of 95°C for 3 s and 60°C for 30 s. Each model was run in duplicate (two independent biological replicates). At each time point, two technical replicate samples were collected from each model, yielding a total of four measurements per time point. 16S rRNA was used as the endogenous control, validated for stability using geNorm across all conditions. Gene expression was quantified using the ΔΔCt method, normalized to 16S rRNA, and adjusted by dividing by log_10_(CFU/g) to account for bacterial load.

Positive controls included ECC strains with constitutive β-lactamase expression induced by cefoxitin. Data were analyzed with QuantStudio software. All qPCR data adhered to MIQE guidelines (57).

## RESULTS

### Baseline MICs and inoculum effect

At baseline, all ECC isolates were susceptible to the tested β-lactams at the standard inoculum (10⁶ CFU/mL) but demonstrated a measurable inoculum effect, with MICs at the high inoculum (5  ×  10⁷ CFU/mL, 100-fold higher) at least ≥8-fold higher (Table 1).

### Pharmacokinetic parameters

Achieved PK parameters (fC_max_, *t*1/2, % fT > MIC) are presented in Table 3. fC_max_ and *t*1/2 values for all simulated regimens were within ±10% of targets.

### Pharmacodynamic models

**Fig 1:**
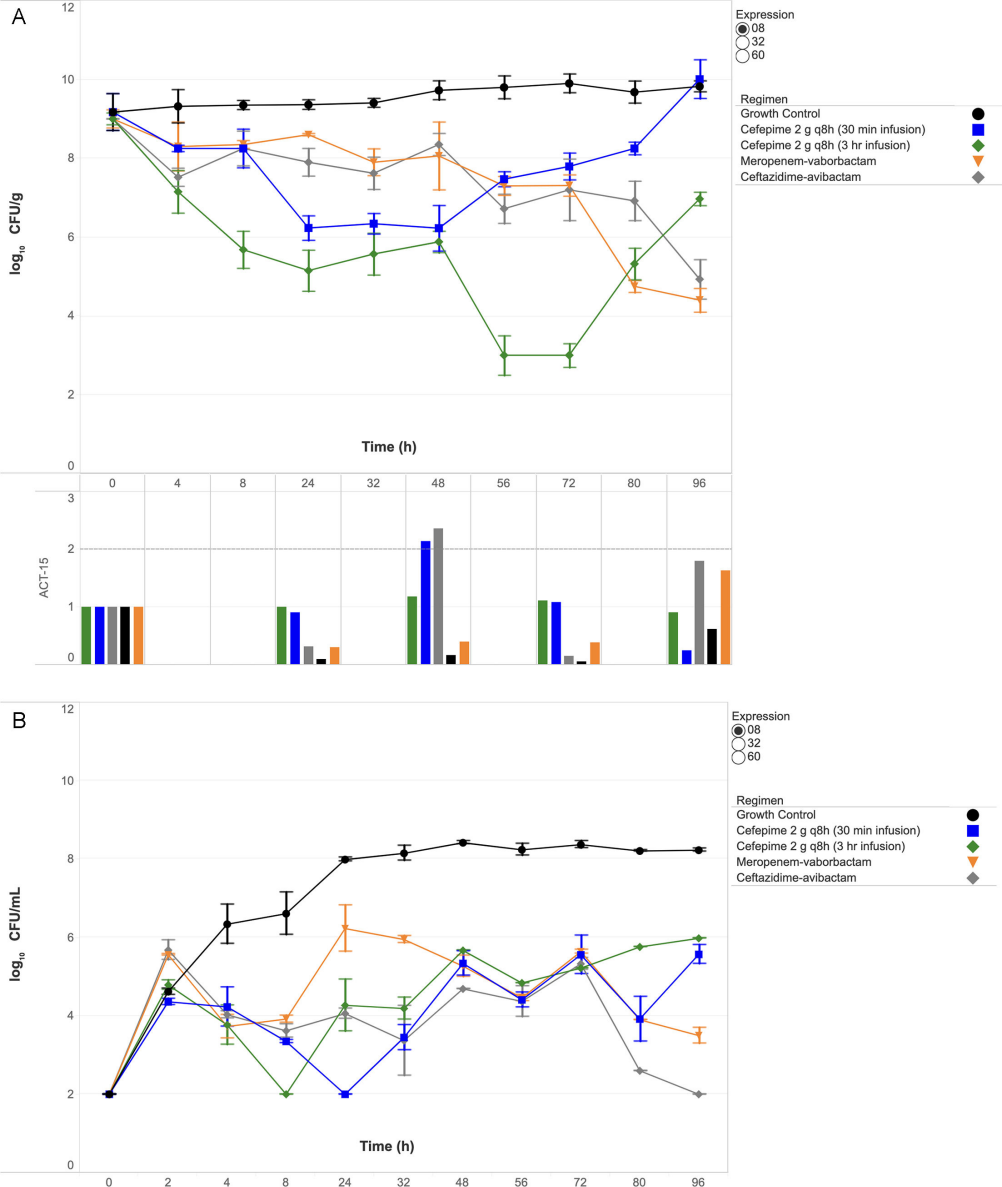
Bacterial burden and gene expression in SEV clots (**A**) and bacterial burden in broth (**B**) against ECC isolate 0008 under humanized antibiotic exposures. Abbreviation: CFU, colony-forming unit. The dashed horizontal line in the gene expression panel indicates the ≥2-fold induction threshold, denoting biologically significant upregulation.

**Fig 2:**
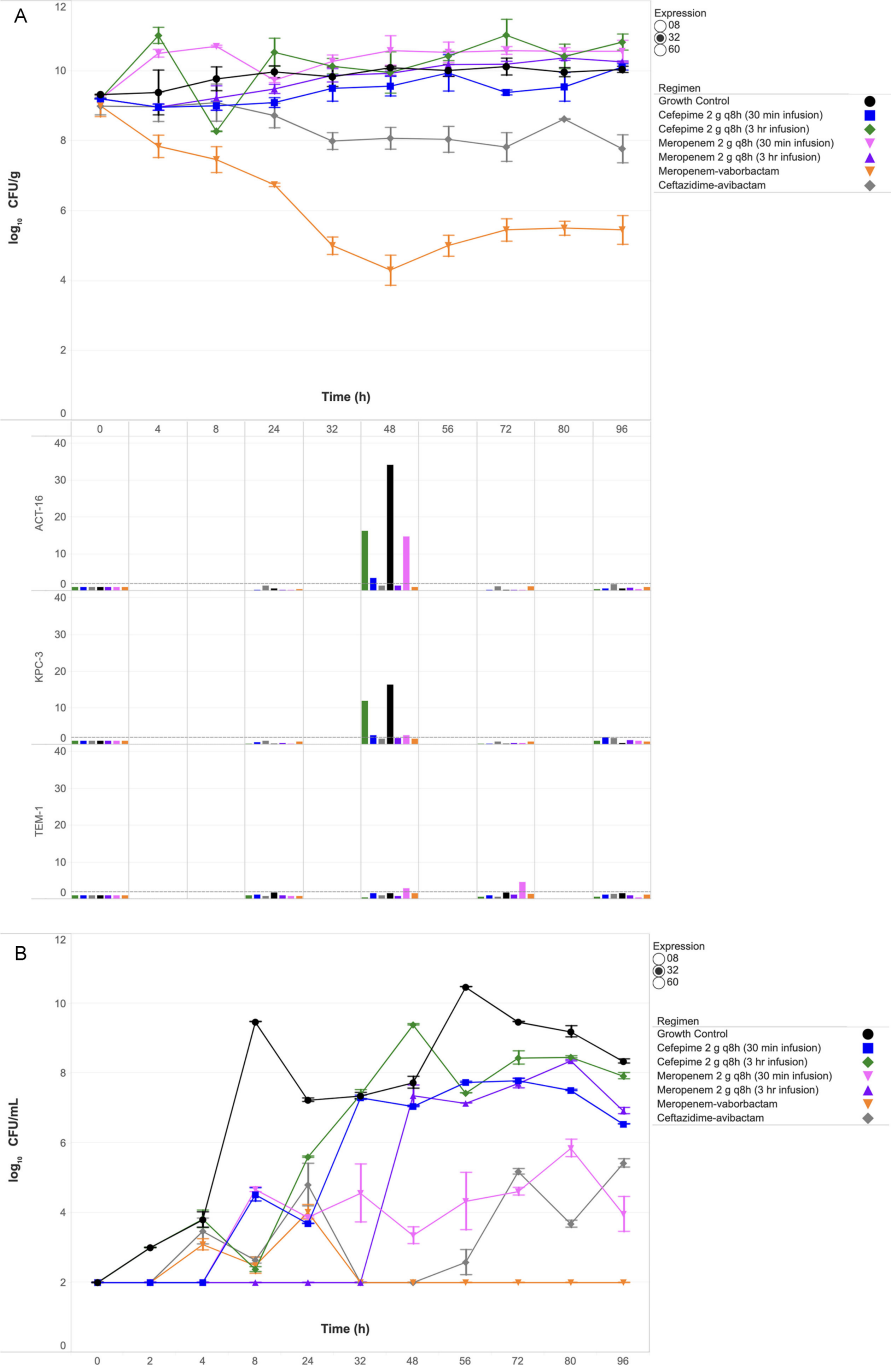
Bacterial burden and gene expression in SEV clots (**A**) and bacterial burden in broth (**B**) against ECC isolate 0032 under humanized antibiotic exposures. Abbreviation: CFU, colony-forming unit. The dashed horizontal line in the gene expression panel indicates the ≥2-fold induction threshold, denoting biologically significant upregulation.

**Fig 3:**
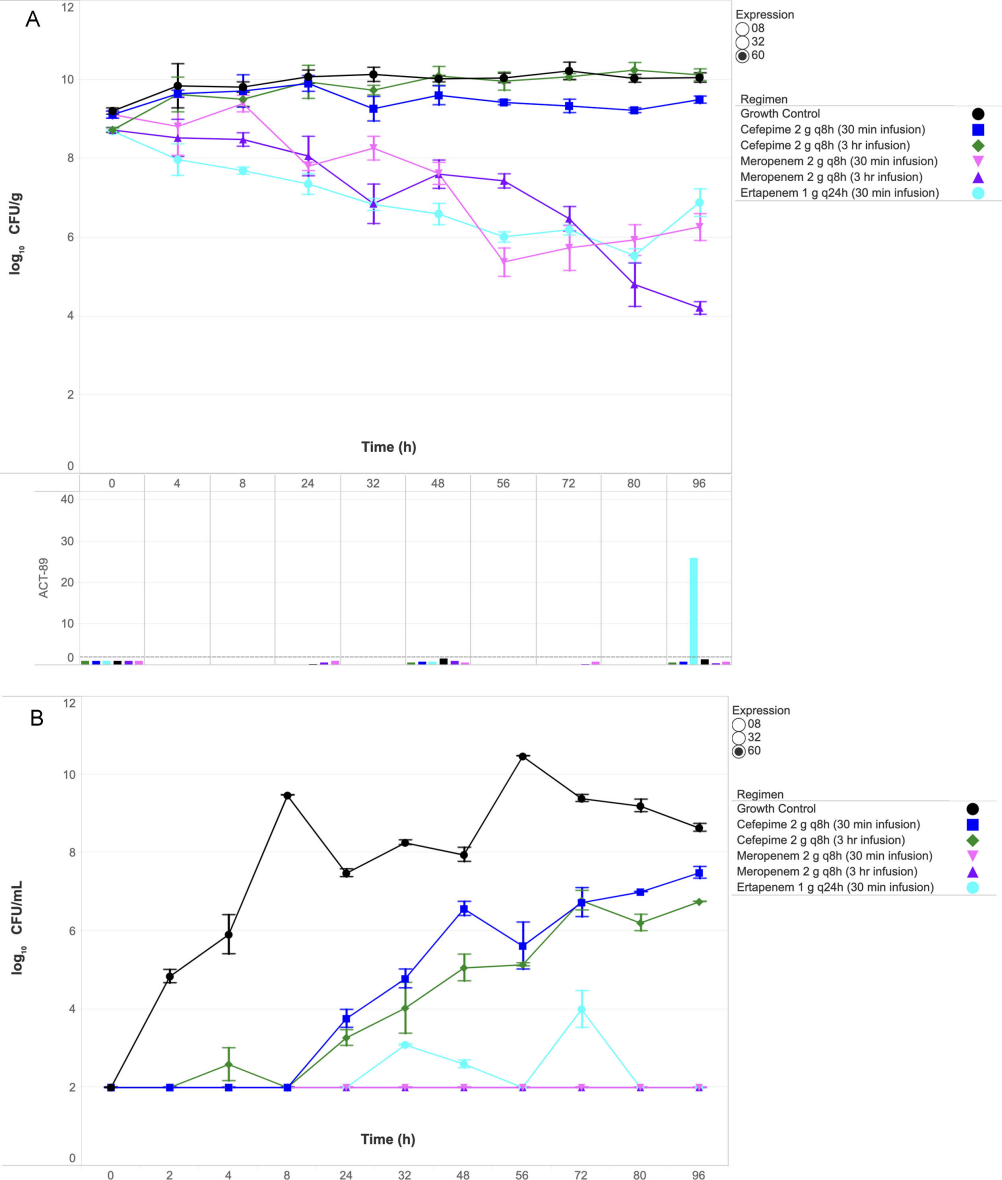
Bacterial burden and gene expression in SEV clots (**A**) and bacterial burden in broth (**B**) against ECC isolate 0060 under humanized antibiotic exposures. Abbreviation: CFU, colony-forming unit. The dashed horizontal line in the gene expression panel indicates the ≥2-fold induction threshold, denoting biologically significant upregulation.

Quantitative changes in SEV and broth bacterial burdens (log_10_ CFU/g and CFU/mL) are shown in Fig. 1 to 3 for isolates 0008, 0032, and 0060, respectively. Key findings for each isolate are detailed below.

### Isolate 0008 (ACT-15)

Cefepime failed to sustain bactericidal activity with either 30 min or 3 h infusions despite initial reductions (Fig. 1A). The 30 min regimen reduced SEV burden by 2.9 log_10_ CFU/g at 48 h but rebounded to a net increase of +0.8 log_10_ CFU/g by 96 h. The 3 h infusion initially achieved greater killing (−6.0 log_10_ CFU/g at 72 h) but also regrew, ending at −2.0 log_10_ CFU/g. Cefepime resistance emerged, with MICs increasing from 8 to >64  µg/mL (Table 4), and ACT-15 expression peaking at 2.14-fold above baseline.

**TABLE 4:** Baseline and post-SEV model MICs^*c*^

Antibiotic regimen^*a*^	Isolate MIC (mg/mL)^*b*^		
	0008	0032	0060
Cefepime (30 min infusion)			
Baseline	8.0	8.0	2.0
T72	32.0	8.0	8.0
T80	32.0	8.0	32.0
T96	>64.0	>64.0	>64.0
Cefepime (3 h infusion)			
Baseline	8.0	8.0	2.0
T72	32.0	8.0	2.0
T80	32.0	8.0	8.0
T96	64.0	32.0	>64.0
Meropenem (30 min infusion)			
Baseline		0.25	≤0.12
T72		4.0	≤0.12
T80		8.0	≤0.12
T96		>8.0	≤0.12
Meropenem (3 h infusion)			
Baseline		0.25	≤0.12
T96		0.25	≤0.12
Ertapenem			
Baseline			0.25
T96			>8.0
Meropenem–vaborbactam			
Baseline	≤0.5	≤0.5	
T80	**2.0**	≤0.5	
T96	**2.0**	≤0.5	
Ceftazidime–avibactam			
Baseline	1.0	≤0.5	
T96	1.0	≤0.5	

^*a*^ Shaded cells represent regimens not tested in the isolate model.

^*b*^ Bolded values are MICs showing ≥2 doubling dilution increases.

^*c*^ MIC, minimum inhibitory concentration.

Meropenem–vaborbactam achieved sustained bactericidal activity (−4.6 log_10_ CFU/g), with MIC increasing modestly from 0.5 to 2.0 µg/mL and ACT-15 expression peaking at 1.6-fold at 96 h. Ceftazidime–avibactam was similarly bactericidal (−4.9 log_10_ CFU/g) and was the only regimen to maintain broth bacterial counts at the detection limit (2 log_10_ CFU/mL) through 96 h (Fig. 1B), with a stable MIC of 1  µg/mL and ACT-15 expression peaking at 2.3-fold at 48 h. Both regimens achieved significantly greater reductions in SEV clot burden compared with cefepime (*P*  =  0.038).

### Isolate 0032 (ACT-16, KPC-3, and TEM-1)

Cefepime and meropenem (30 min and 3 h infusions) failed to achieve bactericidal activity against ECC isolate 0032 (Fig. 2A). The 30 min cefepime regimen resulted in net SEV growth of +0.9 log_10_ CFU/g, while the 3 h regimen increased SEV burden by +1.6 log_10_ CFU/g, with MICs rising to >64 and 32  µg/mL, respectively. β-Lactamase expression was pronounced, with ACT-16 and KPC-3 induction peaking at 3.5- and 2.5-fold (30 min) and 16.1- and 11.9-fold (3 h), respectively. Meropenem showed similar failure, with SEV increases of +1.4 log_10_ CFU/g (30 min) and +1.1 log_10_ CFU/g (3 h), accompanied by a >8  µg/mL MIC shift and ACT-16 induction up to 14.6-fold.

Meropenem–vaborbactam achieved bactericidal activity (−3.6 log_10_ CFU/g) and was the only regimen to maintain broth bacterial counts at the detection limit through 96 h (Fig. 2B), with stable MICs (≤0.5  µg/mL) and minimal β-lactamase induction (<2-fold). Ceftazidime–avibactam reduced SEV burden by −1.2 log_10_ CFU/g and showed modest KPC-3 and ACT-16 induction (~1.7- to 1.8-fold). Both BL/BLI agents significantly outperformed cefepime and meropenem (*P*  =  0.017), and meropenem–vaborbactam demonstrated superior activity compared with ceftazidime–avibactam (*P*  =  0.023).

### Isolate 0060 (ACT-89)

Cefepime regimens failed to achieve bactericidal activity against ECC isolate 0060 (Fig. 3A). The 30 min and 3 h infusions resulted in net SEV growth of +0.4 and +1.4 log_10_ CFU/g, respectively. Cefepime MICs escalated from 2 to >64  µg/mL, while ACT-89 expression remained minimal (< 2-fold).

Meropenem demonstrated infusion-dependent efficacy: the 30 min infusion achieved a non-bactericidal reduction of −2.9 log_10_ CFU/g, whereas the 3 h infusion achieved bactericidal activity (−4.6 log_10_ CFU/g), with broth counts suppressed near the detection limit by 96 h (Fig. 3B). Meropenem MICs remained stable (≤ 0.12 µg/mL), and ACT-89 expression remained minimal (< 2-fold).

Ertapenem achieved bactericidal activity (−3.2 log_10_ CFU/g) through T80 but demonstrated regrowth by 96 h, ending with a net reduction of −1.9 log_10_ CFU/g. The ertapenem MIC increased from 0.25 to >8.0  µg/mL, and ACT-89 expression increased 25.97-fold by 96 h. Both meropenem regimens and ertapenem significantly reduced SEV burden compared with cefepime (*P*  =  0.031), with the 3 h meropenem regimen producing the greatest overall reduction.

## DISCUSSION

This study employed a 96 h ex vivo SEV model to evaluate the PD efficacy of cefepime, meropenem, ertapenem, meropenem–vaborbactam, and ceftazidime–avibactam against high-inoculum (10⁹ CFU/g) ECC, mimicking deep-seated infections such as endocarditis. Bactericidal activity, β-lactamase suppression, and resistance emergence varied by isolate-specific β-lactamase profile (ACT-15, ACT-16/KPC-3/TEM1, and ACT-89), infusion duration, and bacterial density, emphasizing that MIC results at standard inoculum may not accurately predict antibiotic efficacy under high-inoculum conditions.

### Cefepime efficacy and the role of infusion duration

Cefepime failed to sustain bactericidal activity across all isolates, consistent with its known vulnerability to AmpC hydrolysis under high-inoculum conditions (14, 16, 30, 58). Infusion duration modestly affected early killing, but regrowth and large MIC increases occurred in every case. In 0008 (ACT-15), the 3 h infusion temporarily enhanced early killing but did not prevent resistance emergence, suggesting prolonged exposure may only delay the inoculum effect. In 0032 (ACT-16/KPC-3), both infusions failed, accompanied by marked β-lactamase induction, indicating that high inoculum and enzyme diversity can overwhelm cefepime activity. In 0060 (ACT-89), failure occurred despite a susceptible baseline MIC and minimal (<2-fold) β-lactamase induction, suggesting non-enzymatic mechanisms such as porin or efflux changes (16, 59). Although we did not run parallel low-inoculum experiments, our findings align with prior reports showing substantial cefepime inoculum effects, especially for SDD isolates (17). Similar to previous work where >60% of AmpC hyperproducers showed ≥8-fold MIC increases at high inoculum, our results underscore that standard MIC testing may overestimate cefepime efficacy in high-burden ECC infections and limit its role as a carbapenem-sparing option (14, 16, 59, 60).

### Meropenem and ertapenem regimens

Meropenem efficacy varied by isolate and infusion strategy, reflecting the interplay of PK/PD and resistance mechanisms. In isolate 0060 (ACT-89), the 3 h infusion achieved sustained bactericidal activity, reducing SEV burden by 4.57 log_10_ CFU/g at 96 h with stable MICs (≤0.12 µg/mL), outperforming the 30 min infusion, which initially reduced burden but rebounded by 96 h. Both regimens achieved near 100% fT > MIC. These findings align with clinical recommendations supporting prolonged carbapenem infusions in resistant infections (2, 33, 51).

In contrast, both 30 min and 3 h meropenem infusions failed against isolate 0032 (KPC-3/ACT-16). The 30 min regimen was associated with marked ACT-16 upregulation (14.64-fold at 48 h) and MIC elevation from 0.25 µg/mL to greater than 8 µg/mL, reflecting high-inoculum-driven KPC- and AmpC-mediated resistance. Although the 3 h infusion reduced β-lactamase induction (ACT-16: 1.30-fold, KPC-3: 1.82-fold), bacterial growth persisted, demonstrating that infusion extension alone could not overcome carbapenemase-mediated resistance. These results align with Infectious Diseases Society of America (IDSA) recommendations, which caution against meropenem monotherapy for carbapenemase-producing carbapenem-resistant *Enterobacterales* and favor BL/BLI combinations such as meropenem–vaborbactam and ceftazidime–avibactam, which are associated with improved clinical outcomes and lower-resistance emergence risk (2).

Ertapenem initially achieved bactericidal activity in 0060, but regrowth occurred by 96 h, coinciding with marked ACT 89 induction (25.97-fold) and MIC increases from 0.25 µg/mL to greater than 8  µg/mL, contrasting with meropenem-stable MIC profile. This underscores ertapenem's narrower spectrum and greater vulnerability to AmpC hydrolysis compared to meropenem (6, 61). Together, these isolate-specific responses highlight how high bacterial density and enzyme expression can drive resistance beyond what standard MIC testing at standard inoculum reveals, emphasizing the need for optimized PK/PD dosing strategies and combination therapies for high-inoculum ECC infections.

### β-Lactamase inhibitor combinations

Meropenem–vaborbactam demonstrated the most consistent activity, achieving sustained bactericidal reductions in both 0008 (−4.6 log_10_ CFU/g) and 0032 (−3.6 log_10_ CFU/g), with broth suppression to the detection limit in 0032 and stable MICs (≤0.5 µg/mL). ACT-15 expression peaked modestly (1.6-fold at 96 h) in 0008, while KPC-3 and ACT-16 induction was minimal in 0032, consistent with effective KPC-3 inhibition and partial AmpC suppression (62).

Ceftazidime–avibactam showed mixed efficacy, achieving bactericidal activity in 0008 (−4.9 log_10_ CFU/g) with broth clearance and a stable 1 µg/mL MIC despite a 2.3-fold ACT-15 induction. However, in 0032, it produced only a −1.2 log_10_ CFU/g reduction, with broth growth and modest increases in KPC-3 and ACT-16 expression (~1.7- to 1.8-fold). This limited efficacy aligns with clinical observations where ceftazidime–avibactam resistance emergence is more common, particularly in KPC-producing isolates with enzyme mutations such as *bla*KPC-3 and D179 variants (63).

Our findings support IDSA guidance recommending meropenem–vaborbactam and ceftazidime–avibactam as preferred treatments for KPC-producing *Enterobacterales*, with a slight preference for meropenem–vaborbactam at high bacterial inocula due to its enhanced bacterial killing against KPC-3-producing ECC 0032.

### Limitations of MIC testing and resistance dynamics

The outcomes observed in our high-inoculum model suggest that standard MIC values may not reliably predict antibiotic efficacy, as shown by the divergent responses of isolates 0008 and 0032 despite both having baseline cefepime SDD MICs of 8 µg/mL and by the failure of meropenem (0.25 µg/mL) against 0032 in the context of KPC-3/ACT-16 induction.

Clinical laboratories rarely identify AmpC variants or TEM-1, complicating empiric therapy, especially when high bacterial burdens (10⁹ CFU/g) alter drug activity (15, 21, 64). The differences observed between broth and SEV outcomes, such as ceftazidime–avibactam clearing broth in 0008 but not 0032, or meropenem suppressing both compartments in 0060, suggest that resistant subpopulations may be present but escape detection in standard assays.

In isolate 0060, low ACT-89 expression despite regrowth with cefepime and ertapenem may indicate non-β-lactamase activity, such as porin loss or efflux pump overexpression (e.g., AcrABTolC), though this remains speculative and is supported primarily by prior studies (14). In 0032, meropenem failure despite a stable baseline MIC (0.25 µg/mL) aligns with KPC-3 activity but could also involve porin alterations or efflux, consistent with reports of KPC-3 instability (65–68). Variability in β-lactamase induction (e.g., ACT-16 reaching 16.14-fold in 0032 during cefepime failure vs <1-fold in 0060 with meropenem) may reflect the emergence of resistant subpopulations or dynamic model-induced changes. The absence of genomic and real-time transcriptional analyses limits our ability to fully characterize these mechanisms.

### Clinical implications and guideline context

Retrospective studies have linked cefepime and carbapenem monotherapy to higher mortality in deep-seated gram-negative infections (69), paralleling our observation of cefepime failure across isolates and the limited activity of meropenem against the KPC-producing isolate 0032. Current guidelines recommend 6 weeks of combination therapy (β-lactam with aminoglycoside or fluoroquinolone) for gram-negative endocarditis (class IIa, level C) (70), with cefepime preferred over ceftriaxone for high-burden cases in susceptible isolates. Our findings, although limited to a small number of isolates, underscore the challenges of cefepime and the variable efficacy of meropenem while highlighting meropenem–vaborbactam as a possible alternative. These results support further investigation of combination regimens to address resistant subpopulations in KPC or AmpC contexts, as suggested by synergy studies (71, 72). The reduced activity of ceftazidime–avibactam in 0032, where KPC-3 and AmpC are co-expressed, suggests that dual BL/BLI combinations or adjunctive strategies may be necessary for similar isolates. The broth component of our SEV model, intended to approximate systemic bacterial control, offers a useful framework for considering therapeutic adjustments in high-inoculum infections.

### Limitations

This study provides PD insights but has limitations. The ex vivo SEV model does not replicate host immune responses, tissue penetration, or bacterial metabolic adaptations, which may affect *in vivo* relevance. Our focus on β-lactamase-mediated resistance excludes other mechanisms, such as porin changes or efflux pumps, which could play a role in isolates like 0060, where ACT-89 induction was low despite regrowth. Mechanistic interpretation is further limited by the absence of genomic and transcriptomic data. The use of fixed antibiotic regimens, without dose optimization or combination therapy (e.g., β-lactam with aminoglycoside), may limit extrapolation to varied clinical scenarios. While premodel MICs were determined at both standard (10⁶ CFU/mL) and elevated (5 × 10⁷ CFU/mL) inocula, post-model testing was performed only at standard inoculum, potentially underestimating resistance shifts occurring at the higher densities modeled here (e.g., from 8 to >64 µg/mL in 0032). Antibiotic concentrations were measured in central compartment broth samples and may not fully represent drug levels within the SEV clots. Accurate assessment of drug penetration throughout the vegetations would require specialized methods such as radioactive labeling or spatial distribution analyses, which were beyond the scope of this study. Gene expression was measured directly from bacteria in SEV clots without regrowth to capture dynamic responses under antibiotic exposure over time. As a result, expression variability reflects heterogeneous bacterial states within the model rather than synchronized exponential-phase growth and should be interpreted accordingly.

### Future directions

Future studies should integrate genomic and transcriptomic analyses to explore uncharacterized resistance mechanisms, including porin mutations (e.g., *ompK36*, *ompF*, and *ompC*) and efflux pump overexpression (e.g., AcrAB-TolC). Whole-genome sequencing may identify β-lactamase mutations influencing ceftazidime–avibactam activity, while RNA sequencing could quantify the contribution of efflux systems and AmpC regulatory elements (ampR, ampD, and ampG) to resistance dynamics. Clinical studies evaluating combination therapies supported by real-time qPCR or sequencing to monitor resistance emergence are warranted in high-burden infections. Additionally, PK/PD models encompassing a broader range of inocula (10^6^–10^10^ CFU/g) could better characterize the inoculum effect and inform optimized dosing strategies for ECC endocarditis.

### Conclusion

This study underscores the critical impact of β-lactamase expression, inoculum effects, and infusion duration on β-lactam efficacy in high-inoculum ECC infections, as well as the limitations of relying solely on standard inoculum MIC-based predictions. While cefepime was consistently ineffective, meropenem and meropenem–vaborbactam demonstrated variable activity dependent on resistance profiles. Implementation of advanced diagnostics for rapid β-lactamase detection, combined with tailored combination therapies, holds promise for improving treatment outcomes in these difficult-to-treat infections.

## SUPPLEMENTAL MATERIAL

Table S1 aac.01170-25-s0001.docx Primers and probes used for detection of β-lactamase genes.

ASM does not own the copyrights to Supplemental Material that may be linked to, or accessed through, an article. The authors have granted ASM a non-exclusive, world-wide license to publish the Supplemental Material files. Please contact the corresponding author directly for reuse.
