# Supplementary material for: Molecular and pharmacodynamic insights into β-lactam therapy for high-inoculum Enterobacter cloacae complex infections
Source: Antimicrob Agents Chemother. 2025 Sep 22;69(11):e01170-25. doi: 10.1128/aac.01170-25 (PMC12587624; doi:10.1128/aac.01170-25)
Supplement: Table S1 — Primers and probes used for detection of β-lactamase genes. [file aac.01170-25-s0001.docx]

| *bla*ACT-15 |  |
| --- | --- |
|  | Forward Primer    GGGCTATCGTGACGGTAAAG |
|  | Probe    TTTCACGCCATAGGCTTGTGCATC |
|  | Reverse Primer    ATGACCCAGTTCGCCATATC |
| *bla*ACT-16 |  |
|  | Forward Primer    GGGCTATCGTGACGGTAAAG |
|  | Probe    TTTCACGCCATAGGCTTGTGCATC |
|  | Reverse Primer    ATGACCCAGTTCGCCATATC |
| *bla*ACT-89 |  |
|  | Forward Primer    GGGCTATCGTGACGGTAAAG |
|  | Probe    TTTCACGCCATAGGCTTGTGCATC |
|  | Reverse Primer    ATGACCCAGTTCGCCATATC |
| *bla*KPC-3 |  |
|  | Forward Primer    GGTGTGTACGCGATGGATAC |
|  | Probe    CGGCTCAGGCGCAACTGTAAGTTA |
|  | Reverse Primer    CAGCAAGAAAGCCCTTGAATG |
| *bla*TEM-1 |  |
|  | Forward Primer    GGTGTGTACGCGATGGATAC |
|  | Probe    CCATTGCCCGAGGTGAAGTGGTAT |
|  | Reverse Primer    GACGTTAAACACCGCCATTC |

**Supplemental Table 1**. Primers and Probes Used for Detection of β-Lactamase Genes
